# The role of cultural worldviews in predicating gambling risk perception and behavior in a Chinese sample

**DOI:** 10.1002/brb3.2015

**Published:** 2020-12-21

**Authors:** Wen Xue, Zhonglu Zeng, Zuyun Liu, Anthony D. G. Marks

**Affiliations:** ^1^ College of Teacher Education Ningbo University Ningbo China; ^2^ Gaming Teaching and Research Centre Macao Polytechnic Institute Macau Macao; ^3^ Centre for Studies of Hong Kong, Macao and Pearl River Delta Sun Yat‐sen University Guangzhou China; ^4^ Psychology School of Behavioural, Cognitive and Social Sciences University of New England Armidale NSW Australia

**Keywords:** cultural worldviews, gambling behavior, risk perception, smallest space analysis

## Abstract

**Objectives:**

We investigated the relationships between cultural worldviews, gambling risk perception, and gambling behavior with a sample of tourists in Macao.

**Methods:**

Participants were enrolled at famous landmarks and casinos in Macao, China. Data were collected using several instruments to assess an individual's cultural worldviews, gambling risk perceptions, and gambling intentions.

**Results:**

We found that the three‐dimensional solution was valid for the Chinese version of the gambling expectancy scale. Correlational and mediational analyses revealed that the relationship between an *individualistic* worldview and gambling intention was fully mediated by gambling risk perception. Respondents with an *egalitarian* worldview perceived greater risk associated with gambling than those with other worldviews.

**Conclusion:**

These findings demonstrated the important influence of cultural variables on perceived risk and behavior in gambling. Moreover, understanding gamblers’ worldviews could be beneficial for problem gambling interventions. Future research directions and the limitations of the findings were discussed.

## Graphical abstract

We investigated the relationship between cultural worldviews, gambling risk perception, and gambling behavior with a sample of tourists in Macao. Results indicated that the three‐dimensional solution was valid for the Chinese version of the gambling expectancy scale. Correlational and mediational analyses revealed that the relationship between an individualistic worldview and gambling intention was fully mediated by gambling risk perception.

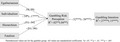

## 1 INTRODUCTION

Gambling, defined as playing a game or betting money with the chance of winning money or another incentive, is a cross‐cultural phenomenon that carries different expressions in different cultures (Custer & Milt, 1985; Raylu & Oei, 2004). One of the issues, however, is problem gambling (PG). Problem gambling is defined as difficulties in spending limited time/money on gambling, and it would result in negative consequences for the gambler or even for the community (SA Centre for Economic Studies, 2005). Previous studies indicated that individuals’ values and judgments were crucial to comprehend gambling behavior. According to Raylu and Oei (2004), cultural values and beliefs played an important role in influencing an individual's gambling behavior. Unfortunately, studies that investigated people's values associated with gambling behavior using an Asian sample are still limited, as most gambling studies corroborating people's values or beliefs were conducted in North American and European countries (Petry, 2005; Raylu & Oei, 2002; Strong et al., 2004).

It is important to complement the current literature by examining individuals’ gambling behavior and values in Asian samples. Studies reported that among Chinese groups, the rate of PG was considerably higher than those other ethnic groups (Lesieur et al., 1991; Oei & Dingle, 2008). Some more specific studies comparing PG rates between Chinese and Caucasian samples also showed similar results, indicating that PG was significantly higher in Chinese samples (Fong & Ozorio, 2005; Shaffer et al., 1999; Wardle et al., 2010). By identifying the relevant values to gambling, the maintenance and development of PG among Asian countries could be understood at an individual level. Moreover, integrated prevention and intervention programs for individuals could be developed effectively. Therefore, it is worth developing a better understanding of people's values in order to explain the development and maintenance of gambling behavior in Asian culture, so that effective intervention or prevention programs can be introduced.

### 1.1 Cultural worldviews

According to Dake (1991, 1992), worldviews could be defined as individuals’ generalized beliefs toward all social relations and the world around them. They functioned as “orienting dispositions,” which governed individuals’ responses when they encountered particular situations (Peters & Slovic, 1996). Similarly, Koltko‐Rivera (2004) summarized that worldviews were a bunch of assumptions about society and physical reality, which powerfully impacted people's behavior and thoughts. Those thoughts reflected their bias toward interpersonal relationships, society, culture, nature, etc. In particular, worldviews also could orient individuals in determining their risk perceptions, and also their preferences in risk management (Leiserowitz, 2006).

Cultural theorists assumed that risks were socially constructed, that is, the preference and depth of fear could vary for every individual in order to maintain their preferred social relation patterns (Douglas & Wildavsky, 1982). Based on this notion, Douglas and Wildavsky (1982) proposed a cultural theory model which described people's preference toward social organizations. The cultural theory model is a two‐dimensional model, containing four main social worldviews. They are as follows:

1. Hierarchism—Worry about social deviance which causes a threat to the current social status quo, but a preference for a clear hierarchical power structure, and a high dependence on experts’ ability to manage hazards.
2. Individualism—A strong belief in social deregulation and the free market, which can provide a high chance for people to maximize their personal benefits, and fear of any restriction on individual autonomy.
3. Egalitarianism—A preference for social equality and justice with a high tolerance for social deviance, a tendency to support consensus‐based decisions and participatory democracy, and a high suspicion of authority.
4. Fatalism—Low levels of social engagement, and a strong belief in destiny, so that any risk management is ineffective.

**FIGURE 1:**
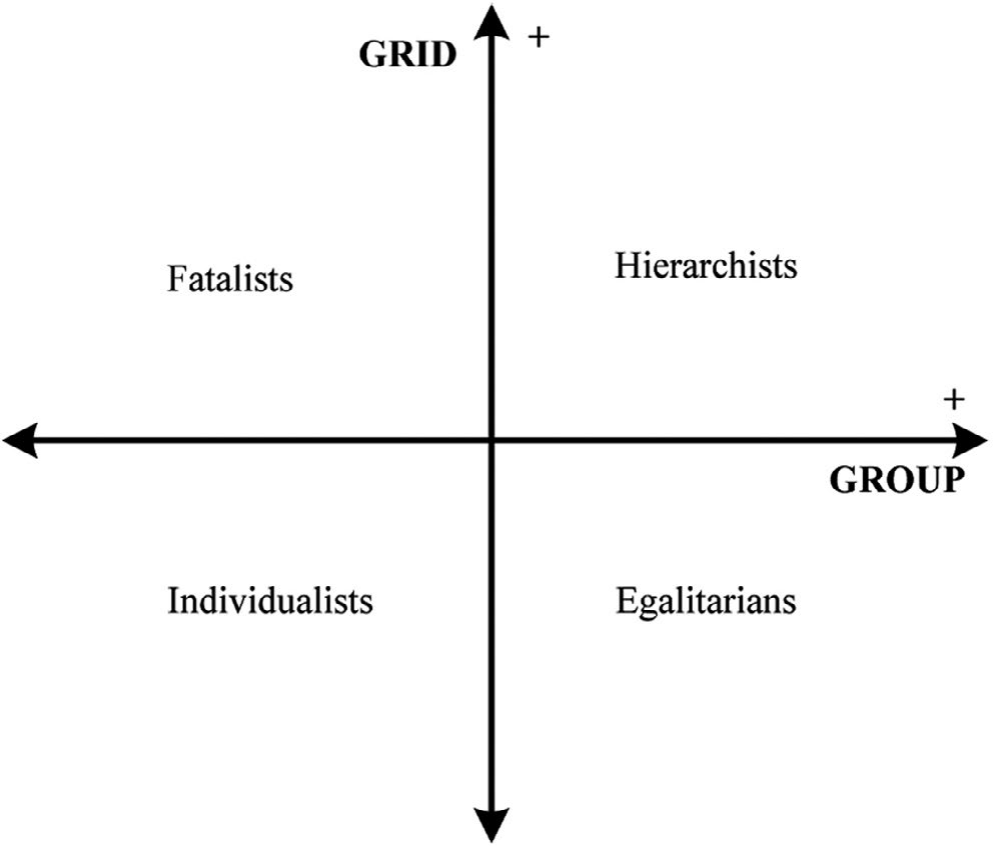
The cultural theory of risk model based on Thompson and Wildavsky (1990)

In this model (Figure 1), dimension “group” is defined as an individual's cooperative relation with others and preference for social bonds, whereas “grid” dimension refers to people's preferred structure of society. Individuals who score high on the “grid” dimension have a preference for a hierarchical social structure, while those with low scores are more likely accept more equal status in spite of class, race, and sex (Xue, Hine, Marks, Phillips, & Zhao, 2016).

Cultural theory implies that people's perceptions about certain risk are generally in accordance with the structure of their preferred social organization (Kahan, 2012). For example, Xue et al. (2014) found those who scored higher on the *egalitarianism* dimension perceived climate change as riskier, whereas fatalists and individualists tended to be more dismissive of environmental risks (Kahan, 2012). Another study revealed certain cultural beliefs were essential predictors of gambling or prolonged PG initiative, which could also change individuals’ gambling patterns and their attitudes toward mental health assistance (Raylu & Oei, 2004).

### 1.2 Measures for cultural worldviews

Dake (1991) developed three subscales to evaluate an individual's commitment to the three dimensions generated by the cultural theory model: *egalitarianism, individualism*, and *hierarchism*. Then in 1992, Dake brought in the fourth subscale to assess the dimension of *fatalism*. Dake's cultural theory scale (CTS) has been widely used for assessing cultural values and has been employed in research exploring the association between different risk perceptions and other variables (Marris et al., 1998; Peters & Slovic, 1996; Sjöberg, 2003, 2004). To date, Kahan (2012, 2007) and Kahan et al. (2007) introduced a revised version of the CTS, with two continuous subscales instead of Dake's four subscales. This new measure is named the cultural cognition scale (CCS), based on a cultural cognition framework. Kahan (2012) believed that the CCS included several advantages over the CTS. When applying Dake's scales, there is a possibility that respondents may score low (or high) on all four subscales. This consequently results in some respondents that may not be categorized into any of these grid/group quadrants defined in Douglas and Wildavsky (1982) model. The CCS tried to resolve this problem by directing respondents to score independently on the dimension “grid” and “group.” That way, each respondent can obtain her/his own set of coordinates in this two‐dimensional space derived from cultural theory. Recently, Xue, Hine, Marks, Phillips, Nunn, et al. (2016) tested all the items from both CCS and CTS together in a representative Chinese sample. They adopted a series of factor analyses to build a new cultural theory measure in the Chinese version, and they validated this new measure with environmental variables, for example, environment‐friendly policy support. The Chinese CTS consists of 12 items, and each set of three items are used to assess one of the four dimensions: *individualism*, *egalitarianism*, *fatalism*, and *hierarchism*.

### 1.3 Gambling and perceived risk perception

People's judgment in a state of uncertainty or risk is an active interdisciplinary research topic in decision‐making studies (Loewenstein et al., 2001). Research has shown that people's perceptions of risky choices can significantly differ by individual (Weber & Milliman, 1997). Studies in many fields have applied the concept of risk perception such as gambling, health care, and climate change, to predict policy preferences and behaviors. A large number of studies claimed that individuals’ values and risk perceptions could result in possible risk‐taking or behavior (Binde, 2009; Breakwell, 2007; Glanz et al., 2008). Investigating gambler's perceived risk can help us better comprehend the pathway and reason for different subgroups of gamblers who confront different gambling‐related hazards (Johansson et al., 2009). An important feature of gambling is that it always involves a risky and uncertain choice and potentially harmful outcomes. When faced with risky options, an individual's risk perception played an important role in determining his or her intention and subsequent behavior (Ajzen, 2011; Breakwell, 2007; Morgan, 2002; Oei & Jardim, 2007; Siegrist et al., 2005). Moreover, a gambler was more likely to engage in a certain risky choice or behavior, according to the risk parameters they perceived (Ajzen, 2011; Weber et al., 2002). Individuals who perceived fewer gambling behavior risks would more likely to engage in gambling behavior or have a higher intention to start. For example, problem gamblers would predict fewer negative outcomes and more benefits with gambling than nongamblers (Derevensky et al., 2010; Wickwire et al., 2007).

Given the large gap between people's beliefs and behaviors, risk perception has been used to explain the indirect effect of worldviews on predicting people's behavior (Xue, Hine, Marks, Phillips, Nunn, et al., 2016). Currently, the literature requires more studies of cooperating cultural worldviews to examine the association between gambling risk perception and gambling behavior.

### 1.4 Worldviews and risk perception

Social scientists have found that people's responses to environmental risks were based on their risk perception (Leiserowitz, 2003), which was governed by their worldviews. People holding different worldviews pay attention to different risks, and their preferences vary. That is, people are profoundly influenced by beliefs and values which defend their preferred pattern of relationships and social status (Wildavsky & Dake, 1990).

Research demonstrated that worldviews were essential predictors of risk perception and choice (Bouyer et al., 2001; Rohrmann & Chen, 1999). Interestingly, individuals who held strong *individualistic* worldviews were more likely to start gambling than others, owing to their higher tendency of risk‐taking (Wildavsky & Dake, 1990). By contrast, people with egalitarian values tended to help other in‐group members (friends or family), who encountered a possibly catastrophic or large loss with PG. This is because they had a high identification with their social relationships and sympathy for group attachment (Rippl, 2002). It is also impossible for *egalitarians* to launch gambling as they have a high tendency to distrust any hazardous or risky activities that might harm the next generation or other group members (Rippl, 2002). It is not clear whether those findings can be replicated in a Chinese sample with regard to gambling. Compared with that, *fatalists* were reported positively to traffic risk perception (Ngueutsa & Kouabenan, 2017), but there is no previous study exploring this cultural belief in gambling risk perception yet. For *hierarchists,* they are more relying on the suggestions from scientists and experts toward any risk management. So, in this study we would also explore the potential associations between *fatalism* and *hierarchism* in gambling risk perception.

### 1.5 Current study

In this study, we translated and validated a gambling risk perception instrument with a Chinese sample. Then, we conducted a mediation analysis to examine whether gambling risk perception could mediate the relationships between cultural worldviews and gambling behavior. We predicted that respondents who had higher scores on the *individualism* dimension would perceive gambling as less risky, so they would have a higher gambling intention. Comparatively, respondents with a higher tendency toward *egalitarianism* were expected to have a lower gambling intention. We also predicted that *hierarchism* and *fatalism* worldviews would have a lower association with gambling risk perception. Last, we also explored the difference in gambling risk perception between gamblers and nongamblers.

## 2 METHODS

### 2.1 Power analysis

No previous study has explored the relationship between cultural worldviews, gambling risk perception, and intention. Therefore, we first ran a *priori* power analysis to determine the minimum number of participants required for the study, using G*Power 3.1.7 (Faul et al., 2009). With five predictors, assuming the target power was 0.90, the median effect size of *f* ^2^ was 0.15 and critical alpha was 0.05, and the result of power analysis suggested the minimum sample size was 116 participants.

### 2.2 Participants and procedure

The Chinese‐version questionnaires were administered in Macao. In total, 400 questionnaires were handed out to tourists from January until April 2018; 364 usable questionnaires were collected. The investigation sites were chosen at Macao's famous casinos (e.g., the Galaxy, Parisian, and Venetian), and other tourist destinations. All procedures performed in studies involving human participants were in accordance with the ethical standards of the Macao Polytechnic Institute research committee (reference number: RP/OTHER‐01/2018) and with the 1964 Helsinki Declaration and its later amendments or comparable ethical standards. The first page of the survey instrument briefly explained the study's purpose and project background. Participants were informed that their responses were anonymous. The participants’ ages ranged from 18 to 73 years, with 54.1% males; 67.7% had full‐time/part‐time work, and 33.9% had completed a college education or more at the time of the investigation.

### 2.3 Measures

The survey instrument consisted of the five parts that follow.

#### 2.3.1 Respondent's demographic information

This section investigated participants’ demographic information such as gender, employment, and education level.

#### 2.3.2 Gambler or Nongambler

One binary item stating that “whether you have gambled before,” to determine whether the participant was a gambler or nongambler.

#### 2.3.3 Cultural worldviews measures

The CTS in the Chinese version was a set of 12 items (Xue, Hine, Marks, Phillips, Nunn, et al., 2016). It is a 4‐point Likert scale, ranging from *I strongly disagree* to *I strongly agree*. Each worldview dimension was assessed by three items, for example, “The government interferes too much in our everyday lives,” “Our society would be better if the distribution of wealth was more equal,” and “We need to dramatically reduce inequalities between the rich and the poor, men and women.” Cronbach's *a* of those four subscales in the Chinese CTS was reported from 0.70 for *individualism* to 0.82 for *egalitarianism*, based on the total sample.

#### 2.3.4 Gambling Expectancy Questionnaire

The third part of the survey assessed the respondent's gambling risk perception. The Gambling Expectancy Questionnaire (GEQ) (Wickwire et al., 2010) had 24 items describing the negative outcomes or potential gains perceived by respondents when performing gambling behaviors. For example, “If I were to gamble, gambling would make me lose a lot of money/lose money/lose and win money the same /win money/win a lot of money.” Respondents with higher scores for each item perceived higher risks in gambling and considered it as a more positive activity. Cronbach's *a* of the GEQ was 0.92.

#### 2.3.5 Gambling intention

The fourth section of the questionnaire evaluated gambling intention. It had 10 items, developed by Moore and Ohtsuka (1997) to assess respondents’ future gambling behavior. It asked respondents about the likelihood that they would engage in the gambling activities listed over the next 12 months, for example, (a) lottery; (b) horses; and (c) poker machine. All the items were assessed on a 5‐point Likert scale (Cronbach's *α* = 0.89).

#### 2.3.6 Potential reasons for gambling

The final section evaluates respondents’ major reasons for repeating a gambling behavior. They needed to select at least one out of eight items, for example, to “Try something new,” and to “Test how lucky I am. ”

### 2.4 Statistical analyses

Exploratory factor analysis was conducted using IBM SPSS Statistical Software (Version 21.0). Correlations were calculated using the *Pearson* correlation coefficients. Then, the confirmatory factor analysis was performed by the HUDAP software (Amar, 2005). Mediation analyses were performed using the AMOS (version 26).

## 3 RESULTS

### 3.1 Factor analysis for the GEQ

Cronbach's *α* for the Chinese version of GEQ without deleting any items was 0.92, suggesting a good level of internal consistency (DeVellis, 1991). An exploratory factor analysis (EFA) was conducted with SPSS software (Version 21) using principal axis factoring. We first applied the eigenvalues‐greater‐than‐one rule (Kaiser, 1960) to the unrotated solution to determine the number of factors to retain. There were four components with eigenvalues greater than 1. However, the result of scree plot suggested a 3‐factor solution instead. Then, we submitted solutions from factors 2–4 to direct oblimin rotations (Δ = 0), to assess each solution's interpretability. As a result, the 3‐factor solution with three distinctive factors was observed to be most interpretable (Table 1). The 3‐factor solution accounted for 59.7% of the GEQ’s total variance. Specifically, the first factor consisted of the three items about material gains designed in the original GEQ scale, and the other items were about other nonmaterial gains, for example, the social status they could obtain (“top of the world”) and “new experience.” So we named this factor as *potential gains*. Factor 2 had items describing the *self and parents’ evaluation*, while factor 3 contained all estimated *negative consequences* from gambling.

**TABLE 1:** Factor pattern matrix for the EFA of Gambling Expectancy Questionnaire

Pattern matrix^a^			
	Factor		
	1	2	3
Better life	0.75		
Earn money	0.70		
New experiences	0.69		
Outlook	0.64		
Money problem	0.64		
Winner	0.58		
Top of the world	0.57		
Parents happy		0.64	
Proud		0.57	
Free		0.55	
Parents proud		0.54	
Self‐esteem	0.50	0.52	
Beat up			0.78
Legal trouble			0.72
Get caught			0.69
Dangerous			0.45

Note

Extraction method: Principal axis factoring. ^a^Three factors extracted. Five iterations required. Items load <0.45 were not reported.

### 3.2 Confirmatory smallest space analysis (SSA) for the GEQ scale

**FIGURE 2:**
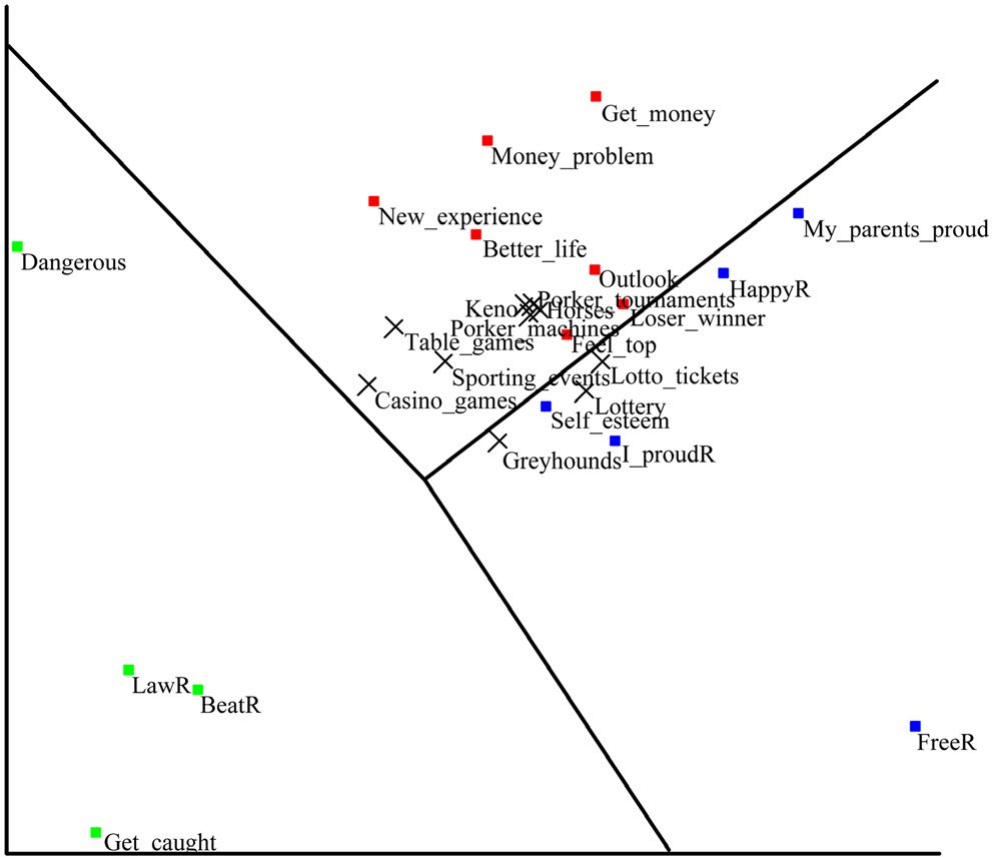
The smallest space analysis plot for the GEQ scale

To assess the validation of the 3‐factor solution suggested by EFA, we performed a confirmatory smallest space analysis (SSA) based on the prediction about the dimensional structure of the GEQ scale in the SSA space through the HUDAP software (Amar, 2005). The coefficient of alienation for the three‐dimensional solution was 0.07, indicating an acceptable level according to Amar (2005). The plot generated by SSA is presented in Figure 2. The pattern of the dimensional relation suggested by the EFA for the GEQ reflected a clear polar structure with three partitions (separation index = 0.90). Then, we submitted those 10 items in the gambling intention as external variables to the spatial configuration generated by GEQ through the HUDAP software. The items’ locations were depicted in the GEQ configuration (see Figure 2). Our hypothesis was supported by the fact that all 10 intention items were in the area between the potential gain partition and the self and parents’ evaluation section, while none of them were located in the negative consequence part. Most gambling intention items were highly related to the items of “Gambling would make me feel like I’m on the top of the world” and “Gambling would make me have a very positive outlook on life.”

### 3.3 Different risk perceptions between gamblers and nongamblers

A correlation coefficient analysis suggested that all the dimensions derived from the GEQ scale were good predictors for an individual's gambling intention for the next 12 months, with the highest effect size of dimension one, potential gains (*r* = .84, *p* < .001). The participant's status (gambler or not) was also highly related to their gambling expectancies, with an effect size from 0.29 to 0.33 of these three GEQ dimensions at a significance level of *p* < .001. Further examination of the risk perception differences between gamblers (*n* = 165) and nongamblers (*n* = 198) was conducted using an independent *t* test. The gamblers reported a higher score on the potential gain dimension (*M* = 2.60, *SD* = 0.77, and self or parents’ evaluation (*M* = 2.66, *SD* = 0.63), but lower in the negative consequences dimension (*M* = 2.74, *SD* = 0.88), whereas the nongamblers reported lower scores for these two dimensions (*M* = 2.05, *SD* = 0.79; *M* = 2.23, *SD* = 0.76), but higher on the negative consequences dimension (*M* = 3.34, *SD* = 0.89). The independent *t* test result of the first dimension was *t*_1_ (360) = 5.58, *p* < .001; for the second dimension, it was *t*_2_ (360) = 4.76, *p* < .001; and for the negative consequences dimension, it was *t*_3_ (360) = 5.22, *p* < .001.

### 3.4 Mediation analysis

**FIGURE 3:**
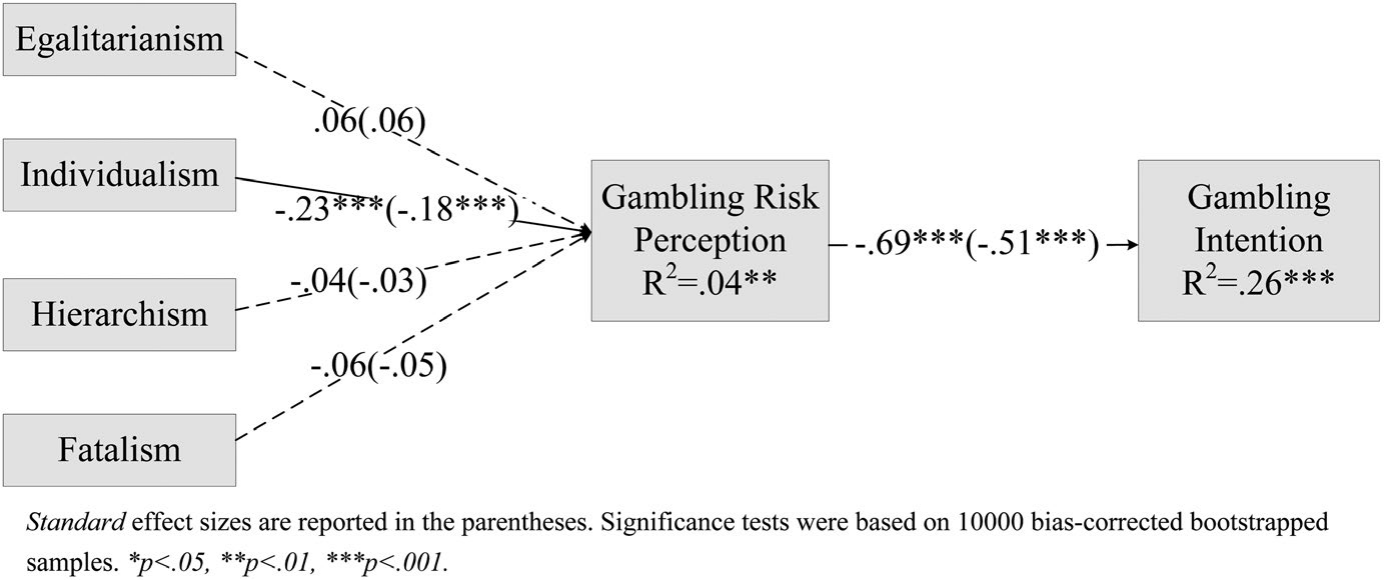
Path analysis model: Gambling risk perception mediating cultural worldview and gambling intention

To test the indirect influence of gambling risk perception in cultural worldviews predicting respondents’ gambling intention, we performed a path analysis using AMOS (version 25). We constructed the mediational path model based on conceptual assumptions with the full sample (*n* = 364) (see Figure 3). The exogenous cultural values were permitted to intercorrelate. Here, we adopted the 10,000 bias‐corrected bootstrapped samples for the significance tests. The data fit the model in an excellent level: The CFI is 1.00, *χ* ^2^/*df* = 0.54, with *χ* ^2^ (12) = 6.46, *p* = .89, SRMR = 0.02, and RMSEA < 0.01, 90% CIs [0.00, 0.02]. As recommended by Kline (2011), the CFI of the model should be greater than 0.90, with χ2/*df* less than 3, SRMR < 0.10, and RMSEA < 0.08 (90% CIs 0.05–0.10). This model significantly explained a 26% variance in gambling intention. As exhibited in Figure 3, *egalitarianism* was the only worldview positively related to gambling risk perception (*β* = 0.06). We also found that *individualism* was the one significantly predicting risk perception in gambling (*β* = −0.18, *p* < .001). However, the worldviews *hierarchism* and *fatalism* were not reliably related to any gambling variables in this model. For the mediation (indirect) effects, we found that gambling risk perception mediated the relationship between *individualism* and gambling intention (*β* = 0.09, 95%, *p*< .001, CIs [0.05, 0.14]). The zero‐order correlation coefficients for all variables are reported in Table 2.

**TABLE 2:** Zero‐order correlation coefficients for all variables

Variable	1	2	3	4	5	6	7	8	9	10	11
1 Gambler/nongambler											
2 Sex	.17***										
3 Employment	.23***	.02									
4 Education	.01	.10	−.05								
5 Residential circumstances	−.08	−.04	.07	−.20***							
6 Egalitarianism	−.09	.05	−.12*	−.08	.11*						
7 Hierarchism	−.06	.01	.05	−.01	.11*	.15***					
8 Individualism	−.13*	−.06	−.06	−.06	.01	.19***	.20***				
9 Fatalism	−.08	−.04	−.06	−.07	.07	.17***	.13*	.17***			
10 Risk perception	−.39***	−.22***	−.01	−.03	.04	−.01	.07	.18***	.07		
11 Gambling intention	−.29***	−.20***	−.01	−.05	.01	−.002	.09	.13*	.06	.51***	
Mean	1.45	1.46	1.84	2.68	1.12	2.84	2.58	2.49	2.26	2.64	3.02
Standard deviation	0.50	0.50	1.32	1.27	0.53	0.65	0.60	0.52	0.58	0.68	0.92
Observed minimum/maximum	1–2	1–2	1–5	1–6	1–5	1–4	1–4	1–4	1–4	1–4.36	1–5
Theoretical minimum/maximum	1–2	1–2	1–5	1–6	1–5	1–4	1–4	1–4	1–4	1–5	1–5

Note

For gamblers or not: 1 = gamblers, 2 = nongamblers, 0 = not disclose/do not know (59 respondents prefer not disclose their identity).

* *p* < .05,

** *p* < .01,

*** *p* < .001.

**FIGURE 4:**
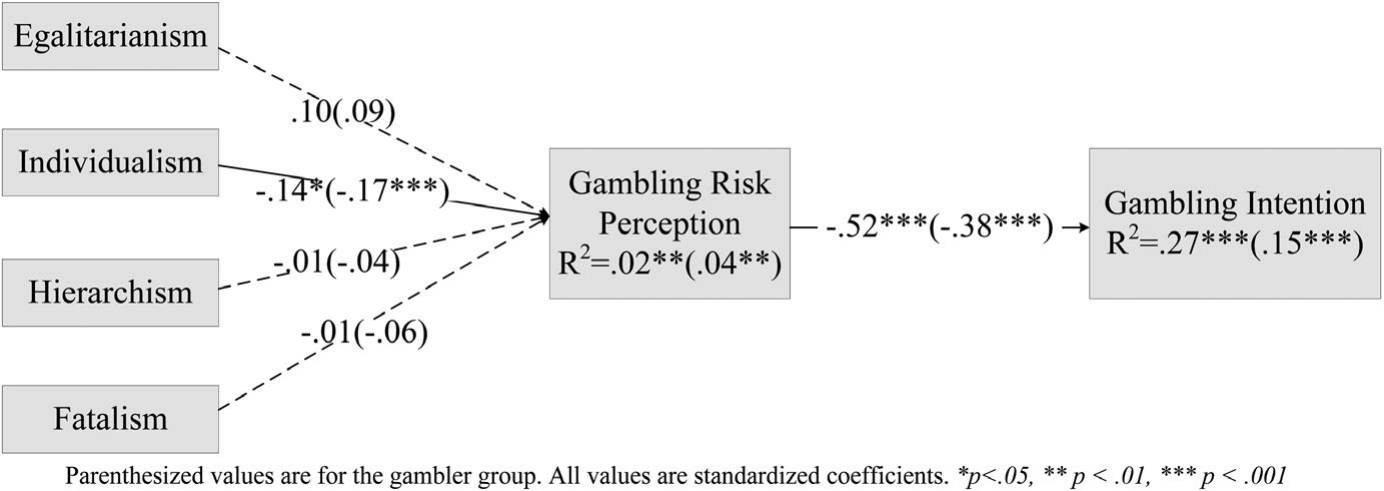
Summary of nongambler/gambler group path model assessing the effect of cultural worldviews on gambling risk perception and gambling intention

To test whether the mediation model we built might vary as a function of gambling experiences, we added a moderation analysis. In this analysis, we divided the full sample into two groups: (a) gamblers (*n* = 165) and (b) nongamblers (*n* = 198). As suggested by the fit indices, both unconstrained and constrained models fit the dataset well. Here, we chose the unconstrained model, where we interpreted the path coefficients for each group separately. Contradictory to our hypotheses, there was no significant difference in these two groups concerning their means on any of the variables included in the path analysis. Figure 4 exhibited all the variance and path coefficients in the two groups. The gambling risk perception and cultural variables explained about 27% of the variance in gambling intention for the nongambler group, but only 15% for the gambler group. In both models, higher levels of egalitarianism and lower scores on individualism predicted more risks associated with gambling, and a higher perceived risk was associated with less involvement in gambling behavior. Focused contrasts comparing the magnitude of the path coefficients between the two groups revealed that risk perception was a significantly stronger predictor of gambling intention for the nongambler group (*β* = −0.52, *p* < .001) than the gambler group (*β* = −0.38, *p* < .001). However, the negative correlation between individualism and risk perception was stronger for gamblers (*β* = −0.17, *p* < .001) than for the nongambler group (*β* = −0.14, *p* < .05).

## 4 DISCUSSION

The study aimed to investigate the role of cultural worldviews in individuals’ gambling behaviors and risk perceptions among a Chinese population. Our findings were partially consistent with the hypotheses. We found that individual's gambling behavior and an *individualistic* worldview were fully mediated by perceived gambling risks. Meanwhile, we found that other worldviews derived from cultural theory were not significantly related to the gambling variables in the model. These results suggested that more applying cultural worldviews to gambling are necessary.

### 4.1 The dimensionality of gambling risk perception

Exploratory factor analyses of the Gambling Expectancy Questionnaire indicated that three factors should be retained, which we labeled in order as *potential gains*, *self*‐ *or parents’ evaluation*, and *negative consequences*. Our three‐factor solution was different from solutions reported in previous research that investigated the GEQ in American samples (Wickwire et al., 2010). In Wickwire and Meyers's study (2010), self‐evaluation and parents’ evaluation were reported as two distinctive factors; this might be because Chinese people evaluated themselves based more on their parents’ evaluation (Lei, 1997). In the confirmatory factor analysis, we found that all of the gambling intention variables were located in the same region between potential gains and self‐ or parents’ evaluation partitions, which suggested that people who believed gambling could bring potential gains and higher self‐evaluation were more likely to gamble. This result was in line with previous research assessing gambling outcomes in predicting gambling behaviors (Stewart et al., 2015). Compared with gamblers, nongamblers scored higher on the negative consequence dimension, but lower on the potential gain dimension. This finding indicated nongamblers had concerns about the negative consequences of gambling and had a less positive attitude toward the gambling behavior rewards than gamblers. In general, Chinese gamblers indicated the main reason for gambling was to achieve a better socioeconomic status. This was similar to other studies with Chinese samples (e.g., Zeng, 2005; Zeng et al., 2009).

### 4.2 Cultural values, gambling risk perception, and behavior

To test the associations between cultural worldviews, gambling risk perception, and intention, we conducted multigroup mediation analyses. The results suggested that the *individualistic* worldview was a strong predictor of respondents’ gambling risk perceptions in both groups. This finding confirmed previous research in the health and pro‐environmental behavior area; individualistic respondents were dismissive to potential hazards or risks than other worldview holders (Kahan et al., 2007; Xue et al., 2014). Meanwhile, previous studies suggested the worldview *egalitarianism* should be positively linked to an individual's health or environmental risk perception (Douglas & Wildavsky, 1982; Peters & Slovic, 1996). However, our findings indicated the prediction from egalitarianism to gambling risk perception was not significant, although these two variables were associated positively. This suggested an egalitarian value might not strongly determine individuals' gambling risk perceptions and behaviors. The other two worldviews were not statistically significant predictors for gambling risk perception, which supported our research hypotheses. For the gambling risk perception, it fully mediated the relationship between gambling behavior and *Individualism*. However, gambling risk perception more significantly predicted gambling intention among nongamblers than gamblers. This could be because gamblers consider positive outcomes more important than negative ones, or they feel the urge to gamble, so they choose to neglect potential negative consequences (Spurrier & Blaszczynski, 2014). We also found that the overall variance in gambling intention explained by gambling risk perception and cultural worldviews was just 15% for Chinese gamblers and 27% for nongamblers. This indicated that between the values and behaviors, some other variables should be considered and added to the model. For example, other cognitive biases such as unrealistic optimism were proposed as an essential factor resulted in gambling and PG (Rogers, 1998; Swekoski & Barnbaum, 2013).

Implications for these results suggested that targeted and integrated interventions for PG may need to consider cultural factors. Future studies should investigate problem gamblers’ worldview, which would be useful to understand their pathway or gambling motivation.

## 5 LIMITATION

A couple of limitations should be considered when interpreting the findings. Firstly, the sample consisted of tourists in Macao (a city similar to Las Vegas) and only about half (52.2%) of respondents reported having engaged in gambling behavior before. Further research is required to explore whether our results are generalizable to other specific samples, for example, clinical samples. Secondly, given that we performed a correlational and cross‐sectional design, inferences about causality should be considered. In the future, more experimental and/or longitudinal studies are needed to demonstrate a correlational level between cultural beliefs and behaviors and if they reflect bidirectional or unidirectional causal effects. Moreover, it would be interesting to investigate the associations between unrealistic optimism, emotional variables, and cultural worldviews among problem gamblers.

## 6 CONCLUSIONS

The present study supported previous findings of the positive relationship between an *individualistic* worldview and risk‐taking. The results of current research also expanded on previously established research by exploring the role of worldviews in people's decision‐making when gambling. Considering the advocacy of public health in China, these results suggested that understanding the targeted gamblers’ worldviews by groups could be a beneficial addition to PG interventions.

## CONFLICT OF INTEREST

The authors declare that they have no conflict of interest.

## AUTHOR CONTRIBUTION

Dr. Wen Xue conceived and designed the work, analyzed the data, and drafted the article. Zeng Zhonglu collected data, performed polishing, and approved the final version to be published. Professor Liu Zuyun critically revised the article. Dr. Anthony D.G. Marks contributed to questionnaire design and collected data.

### Peer Review

The peer review history for this article is available at https://publons.com/publon/10.1002/brb3.2015.

## Data Availability

The raw data used to support the findings of this study have not been made available because the date also forms part of an ongoing study.
